# Role of LncRNA score and PVT1 in primary hyperparathyroidism-- a preliminary study

**DOI:** 10.1186/s40463-021-00509-x

**Published:** 2021-04-28

**Authors:** Dongxue Zhang, Bojun Wei, Long Li, Tao Jiang, Xiaoai Yao, Xin Liu, Yuyan Sun

**Affiliations:** 1 Department of Endocrinology, Beijing Shijitan Hospital, Capital Medical University, No.10 Tieyi Road, Haidian District, Beijing, 100038 China; 2 Department of Thyroid and Neck Surgery, Beijing Chaoyang Hospital, Capital Medical University, Beijing, 100020 China; 3 Department of Otorhinolaryngology, Beijing Shijitan Hospital, Capital Medical University, Beijing, 100038 China; 4 Department of Pediatric Surgery, Capital Institute of Pediatrics, Beijing, 100020 China; 5 Department of Medical Genetics and Developmental Biology, Capital Medical University, Beijing, 100069 China

**Keywords:** LncRNA score, PVT1, Hypercalcemia, Parathyroid cancer

## Abstract

**Background:**

Dysregulated lncRNA score and PVT1 expression may be involved in cancer. However, relationships of lncRNAs with hyperparathyroidism and parathyroid cancer (PC) diagnosis remain mysterious.

**Methods:**

Using quantitative real-time polymerase chain reaction (RT-qPCR), expression profile of PVT1 was evaluated in 57 patients with primary hyperparathyroidism, including 11 with parathyroid cancer (PC) and 46 with parathyroid adenoma (PA).

**Results:**

Higher levels of lncRNA score and PVT1 expression were associated with increased serum calcium level after water ingestion and PC risk (*P* < 0.05). Accordingly, lncRNA score and PVT1 expression were increased with varying degrees of hypercalcemia in PC. A higher level of lncRNA score (but not PVT1) was an independent risk factor of PC, with an AUC up to 0.872 (95% CI: 0.756–0.945, *P* < 0.001). Moreover, lncRNA score was more valuable (with AUC 0.974, sensitivity of 85.71% and specificity of 100%, respectively) than intact parathyroid hormone (iPTH) in predicting risk of PC among patients with hypercalcemia (especially based on greater AUC, *P* = 0.010).

**Conclusion:**

Increased lncRNA score is correlated with an elevated level of serum calcium, which may serve as a potential biomarker for PC diagnosis, especially with hypercalcemia.

**Graphical abstract:**

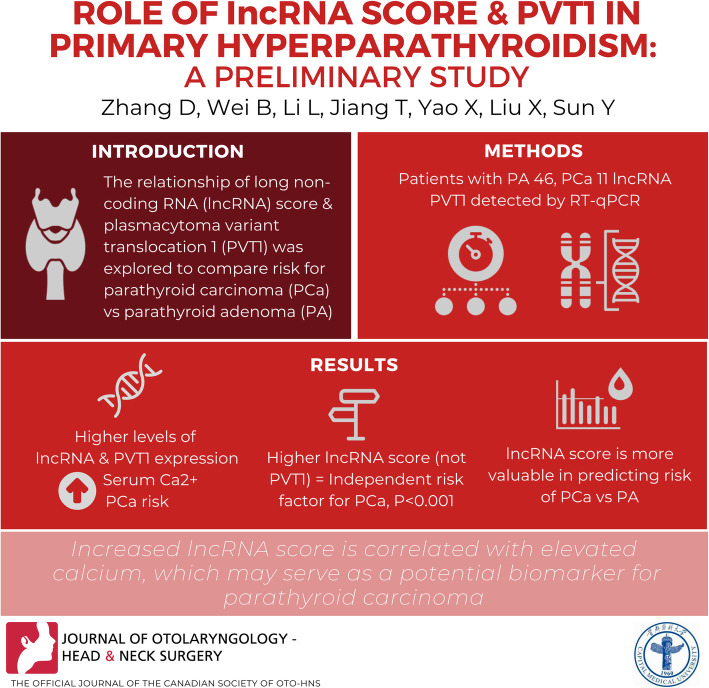

**Supplementary Information:**

The online version contains supplementary material available at 10.1186/s40463-021-00509-x.

## Introduction

Primary hyperparathyroidism (PHPT) is caused by inappropriate secretion of parathyroid hormone (PTH) from parathyroid gland. The estimated prevalence of primary hyperparathyroidism in the USA was 0.86% in 2008–2009 [1]. Familial hyperparathyroidism accounts for 2–5% of total cases. Sporadic PHPT caused by a solitary benign parathyroid adenoma (PA) takes about 80%, hyperplasia with four-gland about 10–15%, and parathyroid carcinoma (PC) about less than 1% [2]. The underlying mechanism of sporadic PHPT is yet to be clarified [2]. In addition, diagnosis of PC remains challenged unless definite lymph node metastasis, or vascular/capsular tumor invasion, or even distant metastasis has been detected [3]. Hence, understanding molecular mechanism is vital for early diagnosis, therapy and prognosis of hyperparathyroidism or PC.

Noncoding RNAs participate in pathogenesis of hyperparathyroidism. For example, miRNAs play key roles in PTH secretion, calcium sensor receptor (CASR) expression and tumorigenesis in PHPT [4]. Long non-coding RNAs (lncRNAs), greater than 200 nucleotides in length, are deregulated in endocrine system disorders, such as thyroid cancer [5–7] and adrenocortical cancer [8]. Our recent study demonstrated that four lncRNAs were involved in hyperparathyroidism [9]. LncRNA score was calculated by the expression levels of LINC00959, lnc-FLT3–2:2, lnc-FEZF2–9:2, and lnc-RP11-1035H13.3.1–2:1. The lncRNA scores were higher in parathyroid cancers than those in parathyroid adenomas. They may participate in hyperparathyroidism. On the other hand, LncRNA plasmacytoma variant translocation 1 (PVT1) also might play a role in parathyroid carcinoma [10]. Hence, we explored potential relationship of lncRNA score and PVT1 with serum calcium level or risk for PC compared to PA among patients with hyperparathyroidism. We also evaluated if lncRNA score might serve as a novel biomarker for diagnosis of PC in Chinese patients.

## Materials and methods

### Patients and samples

This study has been approval by Institutional Review Board (IRB) of Ethics Committees at Beijing Shijitan Hospital and performed according to principles of the Declaration of Helsinki. Written informed consent was obtained from each participant before enrollment.

Parathyroid tissue samples and demographic/clinical information from 57 patients with sporadic hyperparathyroidism were collected and extracted, respectively, during surgical operations at Beijing Shijitan Hospital. All samples were immediately frozen in liquid nitrogen and stored at − 80 °C until use. The patient population consisted of 11 PC and 46 PA patients, including 37 females and 20 males with a mean age of 58.26 ± 12.96 years (range: 24–84). Histopathological diagnosis of PC or PA was confirmed by two pathologists independently. Furthermore, all PC patients had either local invasion, lymph node involvement or distant metastasis. The methods and fasting states differed in maximum calcium detection in different hospitals before visit to our hospital and water ingestion was advised before administration. In hence, to guarantee the comparability of index, all blood samples for biochemical and PTH analysis were collected in the first morning of admission after overnight fasting.

### RNA preparation

Total RNAs were extracted from tissue samples using Trizol Reagent (Invitrogen Life Technologies, Carlsbad, CA, USA) according to the manufacturer’s instructions. In brief, 1 ml Trizol was added to 50 mg tissue sample. After homogenate, tissue samples were incubated with chloroform (200 μl) for 5 min at 20–25 °C. The tubes were shaken by hand for 15 s followed by incubation for 3 min at 15–30 °C. The upper aqueous phase was transferred into another tube after centrifuge at 12,000×*g* for 15 min at 4 °C. Next, isopropanol (500 μl) was added to the supernatant. The mixture was centrifuged at 12,000×*g* for 10 min at 4 °C and incubated at 15–30 °C for 10 min. The supernatant was removed followed by wash of RNA pellet with 1 ml ethanol (75%). Then, RNA pellet was subjected to vortex mixing and centrifugation at 7500×*g* for 5 min at 4 °C. The RNA was dissolved in RNase-free water and incubated at 55–60 °C for 10 min. RNA integrity was evaluated using an Agilent 2100 Bioanalyzer (Agilent Technologies, Santa Clara, CA, USA) with RIN ≥ 7.0 and 28 s / 18 s ≥ 0.7. Samples with OD A260 / A280 ratio closed to 2.0, as detected by a NanoDrop® ND-1000 spectrophotometer (Thermo Fisher Scientific, Waltham, MA, USA), were selected to ensure the quality of total RNA. The total RNAs were reversely transcribed into cDNAs with High-Capacity cDNA Reverse Transcription Kit (Applied Biosystems) and stored at − 20 °C until use.

### Reverse-transcription quantitative polymerase chain reaction (RT-qPCR)

LncRNA PVT1 was detected by RT-qPCR. ABI Power SYBR Green PCR Master Mix (ABI, USA) was used on 7900 HT Sequence Detection System (ABI, USA) following the manufacturer’s instruction. Primers for PVT1 were as follows: F 5′-CAAGCCCCACCAAGAGGAT-3′; R 5′-CAAGATGCAGTAGCCTCAGTGAA-3′. Glyceraldeyde-3-phosphate dehydrogenase (GAPDH) was validated as housekeeping gene (F 5′-TGACTTCAACAGCGACACCCA-3′ and R 5′-CACCCTGTTGCTGTAGCCAAA-3′) and served as internal control. Primers for lncRNAs (LINC00959, lnc-FLT3–2:2, lnc-FEZF2–9:2, and lnc-RP11-1035H13.3.1–2:1) identified were significantly) involved in lncRNA score were as we previously described [9]. The qPCR experiments were conducted as follows: 95 °C denaturation for 10 min; 40 cycles at 95 °C for 10 s, 60 °C for 60 s, and 95 °C for 15 s. The relative expression level of PVT1 was analyzed using 2^-ΔΔCT^ method. Each reaction was performed in triplicate independently.

### Statistical analysis

Statistical analyses were conducted with SPSS (Version 17.0, Chicago, IL, USA) or MedCalc Software (Version 15.8, Mariakerke, Belgium). Quantitative data were expressed as Mean ± standard deviation (SD). Differences between means were compared using Student’s t-test or Mann-Whitney U test. Qualitative data were expressed as numbers or percentages. Multivariate analyses were performed by logistic regression models. Predictive values of indices for PC risk among patients with primary hyperparathyroidism were evaluated by Receiver operating characteristic (ROCs) curves using MedCalc. Non-parametric (Hanley & McNeil,1982) ROC analysis (Z test) was used to compare areas under different curves. A *P* < 0.05 was considered as statistically significant. Method for lncRNA score calculation was described previously [9].

## Results

### Expression profile of lncRNA score and PVT1 and their relationship with clinic-pathological parameters in sporadic primary hyperparathyroidism

To explore the association between lncRNAs (lncRNA score and PVT1) and clinicopathological parameters, patients with sporadic primary hyperparathyroidism were divided into two groups according to lncRNA score or PVT1 expression levels (high vs. low). Higher levels of lncRNA score (Table 1, *P* < 0.05) and PVT1 expression (Table 2, *P* < 0.05) were related to a higher level of serum calcium before operation and a higher risk of PC. The relationship between serum calcium and lncRNA score / PVT1 remained significant after adjusted by serum albumin level (*P* < 0.05). Higher PVT1 level was related to higher lncRNA score (*P* = 0.015). There was no difference in localization accuracy of imaging between patients with differed lncRNA scores (Table 1) or PVT1 expression levels (Table 2).

**Table 1:** Clinical characteristics in patients with high vs. low LncRNA scores

Clinical characteristics		Patients with Low lncRNA score (*n* = 29)	Patients with High lncRNA score (*n* = 28)	***P***-value
Age (SD)(years)		56.5 (14.5)	60.1 (11.1)	0.286
Sex (% M)		24.1	46.4	0.068
Disease course (SD) (month)		67.0 (97.5)	74.0 (90.96)	0.784
Cause of first diagnosis (%)	With any discomfort	51.7	75.0	0.100
	discomfort in Bone system	31.0	35.7	0.783
	Nephrolith	17.2	14.3	0.100
	Discomfort in Digestive system	3.5	10.7	0.352
	Thyroid nodular	10.3	14.3	0.706
	Elevated Ca^2+^ level	27.6	21.4	0.760
Clinical manifestation (%)	Bone system	72.4	60.7	0.408
	Nephrolith	55.2	25.0	0.031
	Digestive system	24.1	32.1	0.565
Laboratory examination	iPTH (SD) (pg/mL)	604.6 (624.2)	544.9 (592.3)	0.713
	Ca^2+^(SD) (mmol/L)	2.75 (0.32)	2.98 (0.42)	0.028^*^
	Albumin adjusted Ca^2+^(SD)(mmol/L)	2.73 (0.32)	2.98 (0.45)	0.020^*^
	P (SD)(mmol/L)	0.79 (0.25)	0.89 (0.49)	0.363
	ALP (SD) (U/L)	236.8 (359.0)	121.9 (52.0)	0.099
Imaging location consistent with histopathology (%, n)	Tc-99 m-MIBI	81.0 (*n* = 21)	86.7 (*n* = 15)	1.000
	Contrast enhanced CT	53.3 (n = 15)	88.9 (*n* = 9)	0.073
	Thyroid ultrasound	75.0 (*n* = 24)	66.7 (*n* = 8)	0.440
Histopathology	Maximum diameter (SD) (cm)	2.10 (2.31)	2.48 (1.31)	0.271
	parathyroid cancer (%, n)	6.9	32.1	0.021^#^
lncRNA PVT1 (SD)		0.32 (0.44)	2.72 (4.89)	0.015^*^

*iPTH* Intact parathyroid hormone, *Ca* Serum total calcium, *P* Serum phosphorus, *ALP* Alkaline phosphatase, *SPECT* Single photon emission computed tomography, *Tc-99 m-MIBI* Technetium-99 m methoxyisobutylisonitrate, *CT* Computed tomography, *MRI* Magnetic resonance. ^*^P < 0.05 for Independent Student’s t-test; ^#^*P* < 0.05 forχ^2^ test

**Table 2:** PVT1 expression and clinical characteristics in patients with hyperparathyroidism

Clinical characteristics		Low-PVT1 (***n*** = 29)	High-PVT1 (***n*** = 28)	***P***-value
Age (SD)(years)		59.4 (13.4)	57.1 (12.6)	0.500
Sex (% M)		31.0	39.3	0.585
Disease course (SD) (month)		64.2 (92.3)	78.7 (96.6)	0.576
Cause of first diagnosis (%)	With any discomfort	48.3	78.6	0.028^#^
	discomfort in Bone system	31.0	35.7	0.783
	Nephrolith	13.8	17.9	0.730
	Discomfort in Digestive system	6.9	7.1	1.000
	Thyroid nodular	17.2	7.1	0.423
	Elevated Ca^2+^ level	24.1	25.0	1.000
Clinical manifestation (%)	Bone system	62.1	71.4	0.576
	Nephrolith	48.3	32.1	0.283
	Digestive system	17.2	39.3	0.082
Laboratory examination	iPTH (SD) (pg/mL)	442.2 (482.3)	713.0 (690.5)	0.091
	Ca^2+^(SD) (mmol/L)	2.76 (0.33)	2.97 (0.42)	0.036^*^
	Albumin adjusted Ca^2+^(SD)(mmol/L)	2.73 (0.36)	2.98 (0.42)	0.021^*^
	P (SD)(mmol/L)	0.88 (0.43)	0.79 (0.33)	0.370
	ALP (SD) (U/L)	144.0 (223.3)	218.0 (297.9)	0.292
Imaging location consistent with histopathology (%, n)	Tc-99 m-MIBI	88.9 (18)	77.8 (18)	0.658
	Contrast enhanced CT	64.3 (14)	70.00 (10)	1.000
	Thyroid ultrasound	70.8 (24)	72.2 (18)	1.000
Histopathology	Maximum diameter (SD) (cm)	2.01 (1.34)	2.58 (1.24)	0.103
	parathyroid cancer (%)	0	39.3	0.000^#^
LncRNA score (SD)		37.8 (8.9)	62.0 (46.9)	0.008^*^

^*^P < 0.05 for Independent Student’s t-test; # *P* < 0.05 from χ^2^ test

### Relationship of lncRNA score or PVT1 expression with the risk for parathyroid cancer

**Fig. 1:**
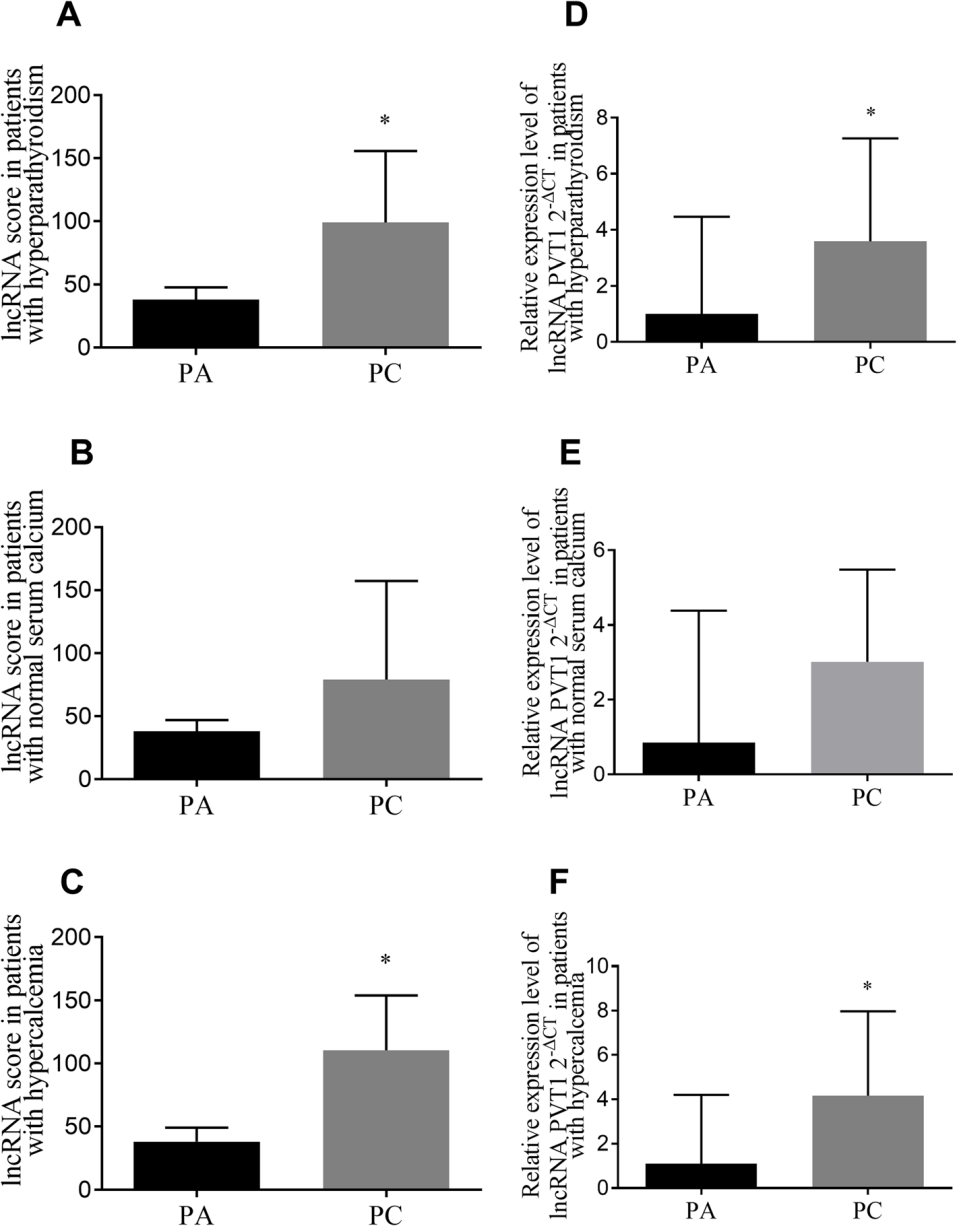
LncRNA score and PVT1 in parathyroid tumors. **a** LncRNA score in PA vs. PC (*P* = 0.000); **b** LncRNA score in PC and PA patients with normal serum calcium (*P* = 0.189); **c** LncRNA score in parathyroid tumors with overall hypercalcemia (*P* = 0.000). **d** Expression levels of PVT1 in PA vs. PC (*P* = 0.002); **e** Expression levels of PVT1 in PC and PA patients with normal serum calcium (*P* = 0.086); **f** Expression of PVT1 in parathyroid tumors with overall hypercalcemia (*P* = 0.001). ^*^P < 0.05 for Mann-Whitney U test

Histological diagnosis for PC in patients with primary hyperparathyroidism remains a critical challenge in clinics. Interestingly, levels of lncRNA score (Fig. 1a, *P* < 0.001) and PVT1 expression (Fig. 1d, *P* = 0.002) were higher in PC than in PA. Expression profiles of lncRNA score (Fig. 1b, c) and PVT1 (Fig. 1e, f) in PC and PA were stratified by different calcium levels. LncRNA score from PC patients was upregulated with hypercalcemia (≥ 2.75 mmol/L) (Fig. 1c, *P* < 0.05). However, there was no significant difference in lncRNA scores between patients with normal serum calcium (Fig. 1b, *P* = 0.189). The expression profile of PVT1 (Fig. 1e, f) was similar to that of lncRNA score. The male gender, intact parathyroid hormone (iPTH) before surgery and higher serum calcium (after adjusted by albumin) were significantly correlated with PC risk (Table 3). In multivariate models, a higher level of lncRNA score remained as an independent risk factor of PC after adjusted for different factors (Table 4). PVT1 was an independent risk factor of parathyroid cancer when adjusted by male, age and iPTH. PVT1 was a risk factor dependent on lncRNA sore (Table 4).

**Table 3:** Demographic and Clinical characteristics in patients with parathyroid cancer (PC) and adenoma (PA)

Parameters	PC (***n*** = 11)	PA (***n*** = 46)	***P***-value
Age (SD) (years)	58.0 (11.6)	58.3 (13.4)	0.871
Sex (% M)	63.6	28.3	0.038^*^
Disease course (SD) (month)	96.3 (135.2)	65.5 (81.2)	0.690
Calcium (SD) (Ca^2+^; mmol/L)	3.11 (0.50)	2.80 (0.33)	0.056
Albumin adjusted Ca^2+^(SD) (mmol/L)	3.15 (0.52)	2.79 (0.35)	0.040^*^
P (SD)(mmol/L)	0.80 (0.42)	0.85 (0.38)	0.551
ALP (SD)(U/L)	150.7 (66.0)	187.5 (291.2)	0.151
Maximum diameter (SD)(cm)	2.89 (1.27)	2.14 (1.29)	0.075
lncRNA score (SD)	99.17 (56.7)	37.8 (10.0)	0.000^*^
PVT1(SD)	3.59 (3.68)	1.00 (3.46)	0.000^*^

^*^P < 0.05 for Mann-Whitney U test; ^#^ *P* < 0.05 for χ^2^ test

**Table 4:** Association between parathyroid cancer and LncRNAs based on multivariate analysis

Parathyroid cancer diagnosis	Model	OR	95% CI	***P***-value
LncRNA score	Model 1	1.099	1.023–1.180	0.009^*^
	Model 2	1.102	1.019–1.192	0.015^*^
	Model 3	1.094	1.021–1.172	0.011^*^
PVT1	Model 1	1.188	1.005–1.405	0.046^*^
	Model 2	1.207	1.021–1.426	0.028^*^
	Model 3#	1.095	1.902–1.328	0.358

Model 1. Adjusted for gender and age

Model 2. Adjusted for gender, age, iPTH, albumin adjusted Ca

Model 3. Adjusted for gender, age, and lncRNA PVT1

Model 3#. Adjusted for gender, age, and lncRNA score

^*^P < 0.05 for logistic regression models

### Diagnostic value of lncRNA score and PVT1 in PC

**Fig. 2:**
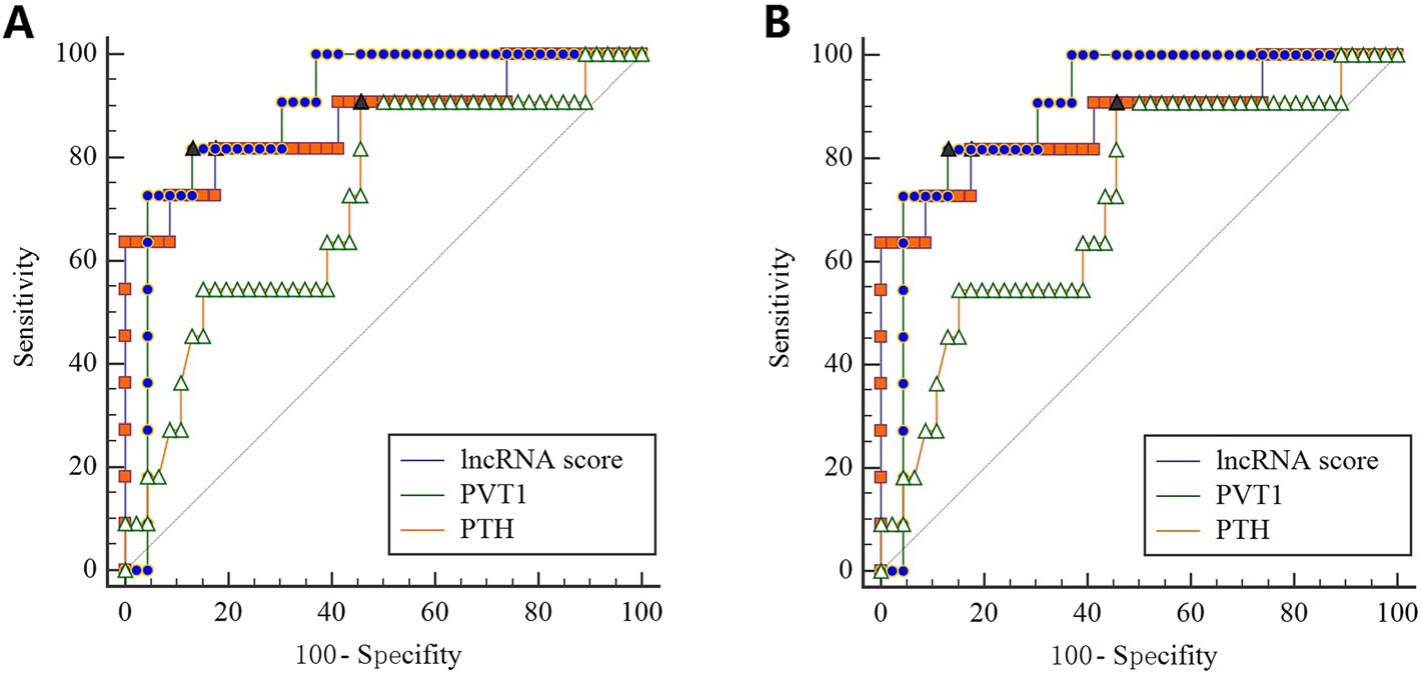
Diagnostic value of LncRNA score vs PVT1 or iPTH in PC. **a** ROC curves of lncRNA score, PVT1 and iPTH in all patients. Area under curve of lncRNA score (*P* = 0.071) and PVT1 (*P* = 0.094) tended to be greater than iPTH. **b** ROC curves of lncRNA score, PVT1 and iPTH in patients with hypercalcemia. Area under curve of lncRNA score (but not PVT1) was significantly greater than iPTH (*P =* 0.010). Black triangular represents for point of Youden-index

Diagnostic value of lncRNA score and PVT1 in PC were evaluated with ROC (Fig. 2a). Area Under the Curve (AUC) of lncRNA score was up to 0.872 (95% CI: 0.756–0.945, *P* < 0.001) with sensitivity of 81.82% and specificity of 82.61%. When lncRNA score greater than 48.2, the accuracy was highest. Moreover, AUC of PVT1 was up to 0.895 (95% CI: 0.785–0.961, *P* < 0.001) with sensitivity of 81.82% and specificity of 86.96%. AUC of iPTH was up to 0.715 (95% CI: 0.580–0.827, *P* = 0.0164) with sensitivity of 90.91% and specificity of 54.35%. AUCs of lncRNA score and PVT1 were tended to be greater than iPTH. However, there was no significant difference among ROCs from lncRNA score, PVT1 and iPHT. Furthermore, AUCs were calculated only from patients with hypercalcemia at the first fasting state after administration (Fig. 2b), because lncRNA score and PVT1 were not dysregulated in patients with normal serum calcium after water ingestion. The AUC of lncRNA score was up to 0.974 (95% CI: 0.836–1.000, *P* < 0.001) with sensitivity of 85.71% and specificity of 100%, respectively. Meanwhile, AUC of PVT1 was up to 0.929 (95% CI: 0.769–0.991, *P* < 0.001) with sensitivity of 85.71% and specificity of 95.45%, respectively. There was no value of iPTH (AUC = 0.711; 95% CI: 0.514–0.863, *P* = 0.088) in PC diagnosis among patients with hypercalcemia. More importantly, AUC of lncRNA score but not PVT1 was greater than that of iPTH in patients with elevated serum calcium after water ingestion (*P =* 0.010).

## Discussion

Primary hyperparathyroidism is a common disorder in endocrine system. Genetic mutations are the leading cause of familial hyperparathyroidism. However, pathogenesis of sporadic hyperparathyroidism remains unclear. In addition, differential diagnosis of PC from PA in patients with hyperparathyroidism is still difficult even after surgery. Many patients with PC have died mainly due to lack of diagnostic biomarker and delay in therapy. Thus, better understanding of biological mechanisms and detecting novel diagnostic and prognostic biomarkers are urgently required for early PC diagnose and management..

In this study, lncRNA score was more upregulated in PC than in PA, which remained significant in patients with hypercalcemia but not those with normal calcium levels. In addition, increased lncRNA score was an independent risk factor of malignancy in multivariate analysis. AUC of lncRNA score was up to 0.872 in all patients. AUC of PVT1 was 0.895, similar to previous report (0.871) [10]. There was no difference between AUCs from lncRNA score and PVT1. Interestingly, a correlation was found between lncRNA score and PVT1 in this study. PVT1 became an independent risk factor when lncRNA score was selected in the multivariate analysis. LncRNA score was still found to be an independent risk factor of parathyroid cancer independent from PVT1. Further, the mRNAs, cis /trans targets of lncRNAs, were also dysregulated in PC based on microarray study [9]. For example, Origin recognition complex 6 (ORC6), upregulated in PC, was predicted as a target gene for lnc-RP11-1035H13.3.1–2:1. ORC6 was involved in cell cycle regulation based on KEGG pathway analysis [9]. In addition, ORC6 might be useful for diagnosis and prognosis of colorectal cancer [11]. Fms-like tyrosine kinase-1 (FLT1), a vascular endothelial growth factor (VEGF) receptor, was predicted to be a target gene of both LINC00959 and lnc-FLT3–2:2. FLT1 was enriched in focal adhesion pathway [9]. Different from other VEGF receptors, FLT1, downregulated in parathyroid cancer [9], was considered as a tumor suppressor gene in choriocarcinoma. LINC00959 and lnc-FLT3–2:2 may participate in parathyroid cancer through downregulating FLT1 [12]. Tumor suppressor fragile histidine triad (FHIT) [13], a target gene for lnc-FEZF2–9:2, was also down-regulated in parathyroid cancer [14]. Meanwhile, in PC patients, Enhancer of zeste homolog 2 (EZH2), a histone methyltransferase, was up-regulated in our previous microarray analysis (Table s1). EZH2 was reported to be involved in sporadic hyperparathyroidism [15, 16]. In addition, EZH2, which was recruited by PVT1, could act as an oncogenic gene in several types of cancer [17–19]. Taken together, PVT1 may participate in parathyroid cancer development through EZH2 pathway. Hence, lncRNA score may be become a diagnostic biomarker for PC.

Here, we found that a higher expression level of lncRNA score was associated with total serum calcium level even after adjusted by serum albumin in the first morning of hospital admission. It has been reported that miRNA-30b level was negatively correlated with serum calcium and iPTH levels in Chinese patients [10]. Noncoding RNAs may play a role in hyperparathyroidism, while the mechanism was still unknown. Lnc-FEZF2–9:2 in the lncRNA score was negatively correlated with serum calcium in the current study. Phosphodiesterase 7 (PDE7), a target gene of lnc-FEZF2–9:2 predicted by a “trans” way, was decreased in PC group in our previous microarray study [9] (Table s1). Silenced PDE7 was reported to reduce the level of 1, 25-dihydroxyvitamin D3 receptor (VDR) [20]. VDR was also down regulated in PCs compared with PAs in microarray study (Table s1). VDR was found to be decreased in parathyroid tumors [21, 22], which may promote parathyroid cell proliferation, set-point for calcium sensing receptor (CaSR) [23], and influence the clinical manifestation of HPT. Taken together, lower lnc-FEZF2–9:2 may participate in hypercalcemia via PDE7-VDR pathway, which also need further molecular study to be proven. In addition, target genes for lnc-RP11-1035H13.3.1–2:1 were clustered in MAPK pathway and decreased in PCs [9]. Disable of CaSR in MAPK pathway activation resulted in acceleration in PTH release and calcium elevation [24]. Lnc-RP11-1035H13.3.1–2:1 may play a role in CaSR dysfunction. Fail in correlation analysis between PTH and lncRNAs may be interferenced by water ingestion before administration. What’s more, increased iPTH concentration was identified in patients with PC than PA in accordance with previous publications, where iPTH levels greater than three times of upper normal limit might be a suspicious sign of PC [25, 26]. Further, lncRNA score, but not PVT1 in this study, was more efficient than iPTH in diagnosing PC among patients with hypercalcemia after water ingestion before hospitalization. Taken together, lncRNA score could act as a potential strategy for parathyroid cancer in patients with hypercalcemia.

Diagnosis of PC remains challenging, although germ-line or somatic mutations in cell division cycle 73 (CDC73) [14] and prune homolog 2 [Drosophila] (PRUNE2) [15] were identified as risk factors for PC. However, these mutations are unable to establish diagnosis of PC [26]. Molecular profile of specific proteins, such as parafibromin, may reveal high-risk features [27], which requires biopsy before surgery [26]. It has been indicated that lncRNAs were amplified in serum or plasma from cancer patients. Dysregulated lncRNAs may serve as a biomarker in peripheral circulation of patients with cancers [7, 28], which indicate the potential of lncRNA score as a plasma marker for pre-operative PC diagnosis.

There are several limitations in this study. First, the roles of lncRNA score in regulating calcium levels and in pathogenesis of hyperparathyroidism were not investigated. Second, potential functions of lncRNA score in carcinogenesis and aggressiveness of PC were not explored. Third, diagnostic value of lncRNA score in serum and tissue sample is guaranteed to be explored in a larger-size prospective cohort of PC patients. Nonetheless, we believed that our study had demonstrated that over-expressed lncRNA score is correlated with an elevated level of serum calcium and may serve as a potential biomarker for PC diagnosis especially in patients with hypercalcemia.

## Conclusion

Upregulation of lncRNA score is correlated with elevated serum calcium. It may serve as a potential biomarker for diagnosis of parathyroid cancer especially in patients with hypercalcemia after water ingestion.

## Supplementary Information

**Additional file 1: Table s1** mRNAs in patients with parathyroid cancer (PC) compared with adenoma (PA) in microarray.

## Data Availability

The datasets used and/or analyzed during the current study are available from the corresponding author on reasonable request.
