# Supplementary material for: Role of LncRNA score and PVT1 in primary hyperparathyroidism-- a preliminary study
Source: J Otolaryngol Head Neck Surg. 2021 Apr 28;50:30. doi: 10.1186/s40463-021-00509-x (PMC8082613; doi:10.1186/s40463-021-00509-x)
Supplement: Supplementary file 1 — Additional file 1: Table s1 mRNAs in patients with parathyroid cancer (PC) compared with adenoma (PA) in microarray. [file 40463_2021_509_MOESM1_ESM.docx]

**Table s1 mRNAs in patients with parathyroid cancer (PC) compared with adenoma (PA) in microarray**

| Target mRNAs | **Fold**  **change** | **Regulation form** | ***P*-value** |
| --- | --- | --- | --- |
| PDE7 | 0.35 | down | 0.016* |
| EZH2 | 4.20 | up | 0.001* |
| VDR | 0.24 | down | 0.028* |

PDE7, phosphodiesterase 7; EZH2, Enhancer of zeste homolog 2; VDR, vitamin D (1,25- dihydroxyvitamin D3) receptor.

* *P* < 0.05.
